# Maintenance of the Austenite/Ferrite Ratio Balance in GTAW DSS Joints Through Process Parameters Optimization

**DOI:** 10.3390/ma13030780

**Published:** 2020-02-08

**Authors:** Bryan R. Rodriguez, Argelia Miranda, David Gonzalez, Rolando Praga, Eduardo Hurtado

**Affiliations:** 1 Corporacion Mexicana de Investigacion en Materiales S.A. de C.V., Ciencia y Tecnologia No. 790, Saltillo 400, 25290 Saltillo, Coahuila, Mexico; brrv92@gmail.com (B.R.R.); eduardohd@comimsa.com (E.H.); 2 Universidad Autonoma de Coahuila, Facultad de Sistemas, Ciudad Universitaria, Carretera a Mexico Km 13, 25380 Arteaga, Coah., Mexico; davidgonzalez@comimsa.com (D.G.); rolandopraga@comimsa.com (R.P.)

**Keywords:** duplex stainless steel, GTAW process, multi-objective optimization, dissimilar joints.

## Abstract

The present work describes the influence of the parameters employed in the gas tungsten arc welding process (GTAW) when nickel powder is used as a filler metal in 2304/2507 duplex stainless-steel dissimilar joints. Multi-objective optimization was applied in order to maintain the austenite/ferrite percentage in the welded zone. A microstructural and phase quantification analysis was performed in each sample through optical and scanning electron microscopes. It was found that a nickel powder addition combined with low heat input increased the biphasic ratio across the different zones of the dissimilar welded samples. Although the austenite volume fraction increased in the 2304 heat-affected zone (HAZ) near to 25%, it was not sufficient according to international standards. The obtained results led to the maintenance of the 50/50 phase percentage in the 2507 HAZ welded joint side, as well as to the increment of the austenite percentage in the 2304 HAZ.

## 1. Introduction

Duplex stainless steel (DSS) derives its name from the stable microstructure at room temperature, which consists of approximately 50% austenite phase (γ) and 50% ferrite phase (δ). This microstructure has a favorable combination of mechanical and anticorrosive properties, ensuring high levels of high-temperature resistance, optimum toughness, and ductility [1,2,3,4].

The welding processes used to join this type of material promote variations to the dual percentage of ferrite-austenite, because the microstructural heterogeneity generated by the thermal gradients can locally generate microstructural changes, influencing the mechanical behavior of the joint [3,4,5,6]. Controlling this dual percentage is of great importance to the maintenance of the mechanical properties, especially of the corrosion resistance. Therefore, different studies have been highly focused on supplemental elements or materials during the welding procedure. In a study conducted by Varbai and Májilinger in 2019, a method to measure the austenite/ferrite (A/F) ratio in the different zones of the welded sample through the analysis of its images using a proper etching attack was studied. Putz et al., in 2019, also studied this topic, focusing on the difference among various methods to measure the A/F ratio on the welds correctly [7,8].

In fusion welding processes of duplex stainless steels, the resultant microstructure in the temperature range to which the material is exposed solidifies as fully ferritic, and for austenite, the transformation cooling rate and the composition are both significant. In multi-pass welding at high temperatures, another phenomenon is observed: ferrite decomposition occurs, causing the formation of harmful secondary phases, such as sigma (σ), chi (χ), carbides, and nitrides phases, which subsequently deteriorate the mechanical and corrosion-resistance properties of the DSS. Santos et al. conducted a study, based on the analysis of thermal history, which showed the temperature measured across the weld and its effect on the microstructural and mechanical behavior, and compared the results with the three-dimensional finite element model built. In addition, Sarlak, Atapour, and collaborators specifically studied the corrosion behavior across the welding in samples obtained by the welding process of a lean duplex stainless steel (LDSS) and its effect on the microstructure [9,10,11].

Various joining techniques, such as plasma, laser, tungsten inert gas (TIG), and gas metal arc welding (GMAW) are being developed to meet the requirements demanded by these materials, avoiding distortion. However, ferritization is a problem with these processes. In addition, the effect generated by the entry of heat into these materials is a research topic. Plasma arc welding has beneficial results for DSS, which is situated at a controlled heat input and an acceptable ferrite ratio compared with other processes. Flux-cored arc welding (FCAW) has gradually attracted strong attention in welding DSS due to its higher welding efficiency compared with the gas tungsten arc welding process (GTAW) process. However, studies on the microstructure, mechanical properties, and corrosion resistance of the DSS FCAW joint are limited. Advanced laser hybrid welding has a great advantage for DSS, because it reduces heat input and distortion level compared with SAW and FCAW [12,13]. The GTAW is a welding procedure that has been employed efficiently in past years to weld materials such as stainless steels due to its facility to produce sound joints that meet specific requirements of industries, added to its facility to work when thin materials are required. However, it has been observed that when the main welding parameters and conditions are not well controlled (welding current, welding speed, shielding gas, etc.), the mechanical and corrosive properties could be reduced. An example of this appears in the heat-affected zone (HAZ) and the fusion zone (FZ), where the microstructure undergoes rapid cooling and heating cycles that result in changes in the orientation and size of the ferritic grain, as well as in excessive amount of ferrite. Previous research reported that austenite contents of less than 30% are inadequate for most industrial applications [14,15,16,17]. Ravisankar et.al. studied the influence of welding speed and power on residual stress during the GTAW process of thin sections with constant heat input: the analysis of the heat source fitting revealed that the heat input of 300 J/mm resulted in a proper weld penetration and HAZ. Chen et.al. analyzed the influence of cooling rates on microstructure evolution and pitting corrosion in the simulated heat-affected zone of 2304 LDSS and found that with resistance in the simulated HAZ of 2304 LDSS, the ferrite phase content increased gradually as the cooling rate increased [18,19].

The constant development of various applications employing duplex stainless steels used in the chemical, petrochemical, nuclear, and energy industries, among others, demands the adaptation of materials that meet the required service conditions, thus, promoting the development of hybrid structures with dissimilar metals (i.e., welding between stainless steels) and combining their respective advantages. However, dissimilar metal welding is often accompanied by a series of problems, such as secondary phase formation, and the distribution of dissimilar metal weld in the fusion zone, which must be controlled [3,20].

Various experiment design techniques (DoE) are currently employed in a variety of applications, with the purpose of identifying parameters associated with a process and determining its optimal configuration to improve process capabilities and performance. One of the most employed DoE techniques is the full factorial design, where the variables’ effect is analyzed for a certain response. The determination of a correct selection by a trial and error method for each study is time-consuming and increases experimental cost; consequently, prediction models are gaining attention because they are employed to enhance parameters through the development of optimizing techniques. In the case of parameters with some randomness, e.g., a manual welding process, a prediction model, such as the general mixed linear model, is proposed [21,22]. When joining materials of similar or variant compositions, the main problem is the selection of welding parameter ranges to obtain the desired properties.

The aim of this investigation is to determine the optimal parameters of the GTAW process that allow an equitable proportion of phases in the microstructure of dissimilar joints to be obtained through multi-objective optimization, considering the employment of variables such as welding current and nickel powder addition.

## 2. Materials and Methods

### 2.1. Materials

UNS 2304 lean duplex stainless steel (LDSS) and UNS 2507 super duplex stainless steel (SDSS) were the base metals (BM) used in this study, with their composition (wt %) shown in Table 1. The base metals were shaped to sizes of 70 × 60 × 3 mm for LDSS and 70 × 60 × 2 mm for SDSS.

Metallographic preparation of the samples was in accordance with standard ASTM E3-11. The samples were etched with modified Beraha’s etch (50 mL HCl, 50 mL H_2_O, 1 g K_2_S_2_O_5_, and 10 g NH_4_ F-HF) for 12 s to reveal the microstructure in the LDSS 2304 side. On the other hand, for the welded zone and the SDSS 2507 side, Marble’s etch (10 g CuSO_4_, 50 mL HCl, and 50 mL H_2_O) was used for 15 s in sequence.

Different characterization techniques were employed to analyze the microstructural behavior of the samples (optical and scanning electron microscopes), in addition to performing a punctual analysis by energy-dispersive X-ray spectroscopy (EDS) to study the A/F percentage in the 2304 HAZ, the 2405-2507 FZ, and the 2507 HAZ of the welded joints.

### 2.2. GTAW Process

**Figure 1:**
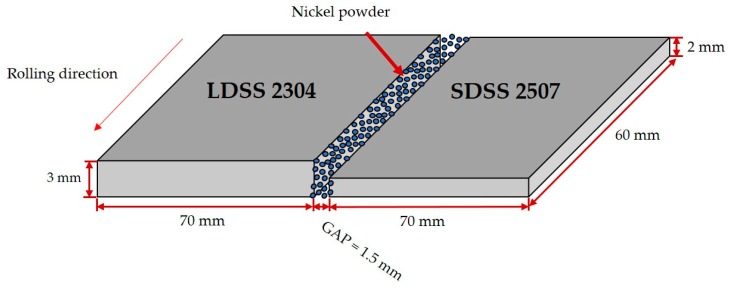
Scheme of the dissimilar duplex stainless-steel butt joint.

The welding procedure was developed employing a Miller Syncrowave 350 CC AC/DC Welder in a rolling direction, after cleaning with acetone to remove any contaminants such as grease and oil. The joint design was in butt configuration with the samples held on a work metal table with a 1.5 mm gap, where 1.5 g of nickel powder with spherical morphology and a diameter range of 150 ± 60 µm was added and impregnated with acetone (see the information in Table 2) as a filler metal before the welding procedure (Figure 1).

The welded joints were performed maintaining the parameters mentioned in Table 3 and were cooled at room temperature. A backing plate was not used in this case.

### 2.3. Welding Parameters

The initial tests were determinant on an experimental design based on a 2^k^ arrange with two factors (current and nickel powder addition) with two levels, which generated four experiments to evaluate (Table 4). The aforementioned was in accordance with the determination of the influence of nickel addition as a filler metal and the welding current variation on the biphasic ratio across the different welding zones of the materials.

**Figure 2:**
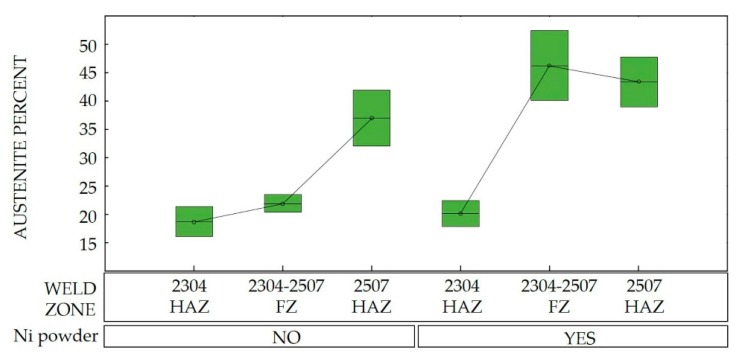
Box plot for austenite percentage. FZ: fusion zone; HAZ: heat-affected zone.

Table 5 presents the obtained results from the statistical analysis. It is important to highlight that both the nickel and the current possessed a significant effect in the austenite increment (value of P < 0.05) in the weld zone and adjacent HAZ to the SDSS 2507. Nevertheless, the statistical results indicated that the previous interaction did not significantly influence the austenite percentage in the LDSS 2304 HAZ. Figure 2 schematizes the aforementioned, in accordance with the box graph of the austenite percentage.

The above led the focus of the study to the random parameter, i.e., the welding speed, because the process is not automated and it is a function of the welder’s ability.

In order to determine the effect of the welding speed, a 2^k^ factorial design with central points was proposed in which the current welding values were considered. Nickel was also added to all the samples. Nevertheless, two central points were added, which are shown in Table 6.

The arc voltage was not contemplated in the experimental design; however, its values are presented in Table 6. The voltage was generated as a function of the established welding current in the equipment, because the welding equipment has a synergic control.

### 2.4. Optimization

Analysis of the results of the above-mentioned testing determined the relation of the process parameters whilst maintaining the equitable proportion of austenite-ferrite in the welded regions. This led to the employment of a mixed-effect model, because regression models only contain error as a random element. However, different situations require models such as the mixed one, where more than one arbitrary term exists. These models are used to describe the relations between an output variable and a few co-variables grouped in accordance with one or more classification factors. This model employed the welding speed result as a non-controllable variable, i.e., it is identified as an arbitrary factor, such as the weld of these materials by the manual GTAW process. In this proposal, current and welding speed functions were used as input variables, respectively, which were correlated with the heat input.

The general mixed lineal model is given by

Y = Xβ + Zv + ϵ(1)

where *x* is a dimension matrix of *N* x *p* known, *β* is a *p* x *1* vector of fixed parameters, *Z* is an *N* x *q* matrix of constant known, *v* is a *q* x *1* vector of arbitrary effects, and *ε* is an *N x 1* vector of arbitrary errors. Assuming that

(2) ~εvN{(00),[G00R]}

where *G* and *R* are known matrices that depend on an unknown parameter *θ* and considering that

Var (Y) = ZGZ’ + R,(3)

models not only imply the *β* parameters but also the variance of the *θ* parameters. Moreover, not only the hypothesis test of the *β* can be observed, but also of the *θ* considering the expression [21,22,23].

(Y-Xβ)’V_Y_^(−1)∗(Y-Xβ) (4)

Once the model that correlated the variables of the process was obtained, a multi-objective optimization supported by a genetic algorithm was developed to determine the values of the parameters that could maintain the austenite ratio percentage equitable in the weld zone and heat-affected zone of the SDSS 2507, increasing the HAZ adjacent to the LDSS 2304 (Table 7).

## 3. Results

### 3.1. Base Metal

**Figure 3:**
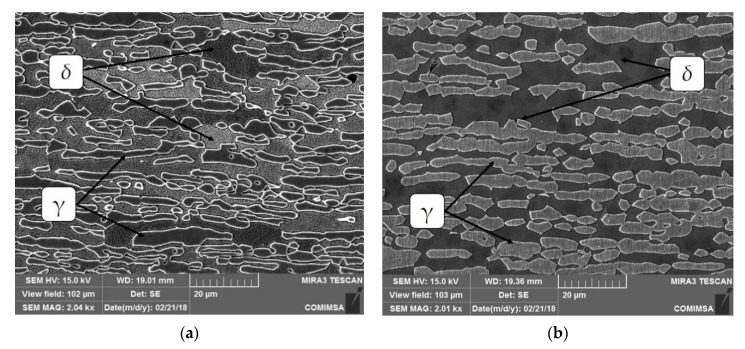
Base metal microstructure observed by scanning electron microscope (SEM): (**a**) SDSS 2304 and (**b**) SDSS 2507. δ: ferrite phase; γ: austenite phase.

Figure 3 shows micrographs of the transversal phase of duplex stainless steel. It is possible to observe a ferritic matrix (δ) in dark color and austenite islands (γ) dispersed across the matrix in light color in both micrographs.

### 3.2. Preliminary Tests

**Figure 4:**
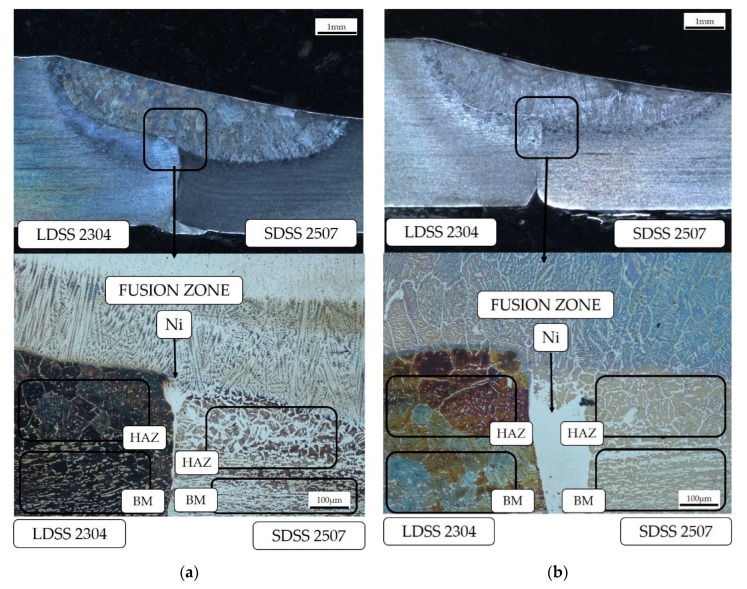
Welded zones by optical microscope to 10 ×: (**a**) Test #3 and (**b**) Test #1.

Through microstructural analysis, it was observed that the joints with Ni powder presented islands with a high content of this element in the interface between the two metals below the fusion zone (Figure 4).

**Figure 5:**
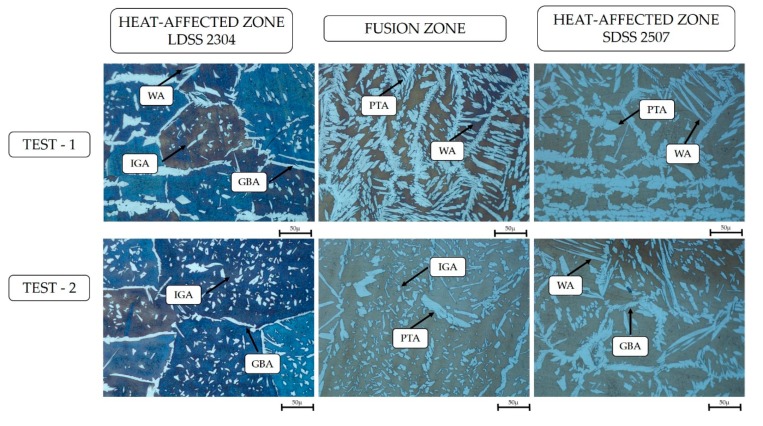
Welded zones by optical microscope to 50 × : 130 A welding current.

**Figure 6:**
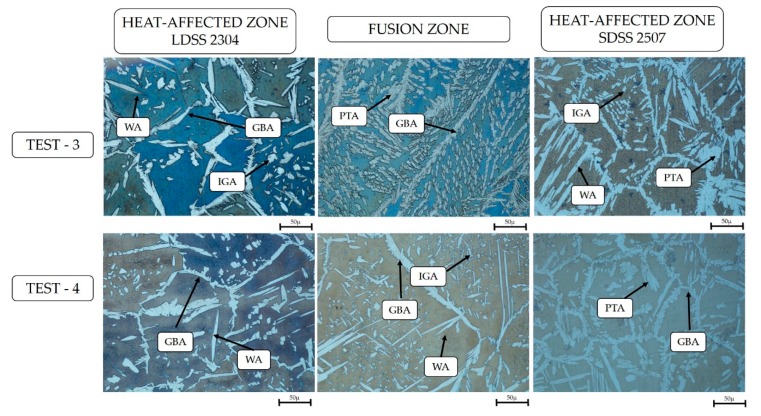
Welded zones by optical microscope to 50 ×: 220 A welding current.

In accordance with the investigations, it was possible to observe different types of austenite produced by the subsequent transitions of the solid-state ferrite phase, which solidified through the melted metal: grain boundary austenite (GBA), Widmanstätten austenite (WA), intragranular austenite (IGA), and partially transformed austenite (PTA) [24] (Figure 5 and Figure 6).

There was a significant difference in the A/F percentage both in preliminary and validation tests when varying the welding speed and Ni addition but not in the morphology of the current phases.

**Figure 7:**
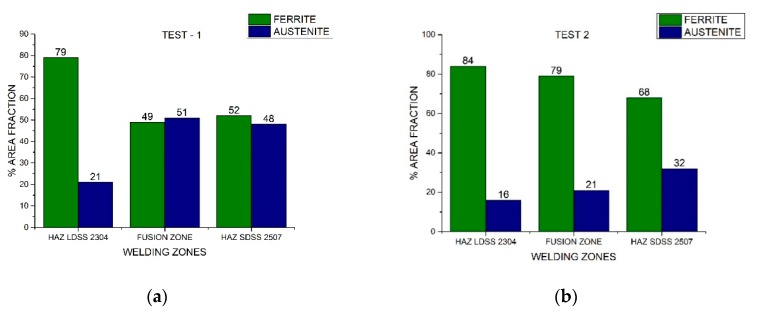
Area fraction through the distinct welded zones: (**a**) Test #1 and (**b**) Test #2.

**Figure 8:**
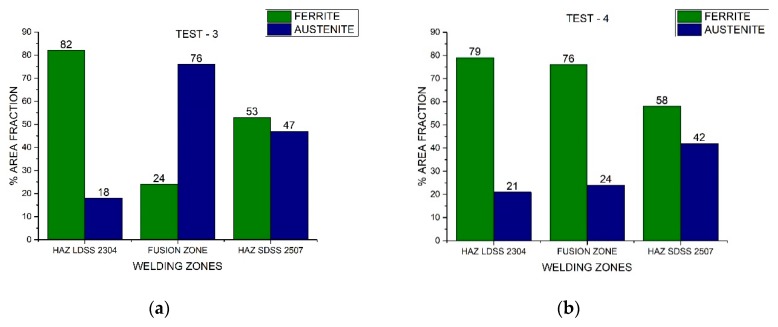
Area fraction through the distinct welded zones: (**a**) Test #3 and (**b**) Test #4.

The area fraction of each phase of both duplex stainless steels through the distinct welded zones is presented in Figure 7 and Figure 8. Nickel absence reduced considerably the austenite percentage in the FZ, while the welding current variation influenced the austenite fraction in the HAZ. The area fraction was directly proportional to the volume fraction.

### 3.3. Tests Joints

#### 3.3.1. Microstructural Analysis and Phase Quantification

**Figure 9:**
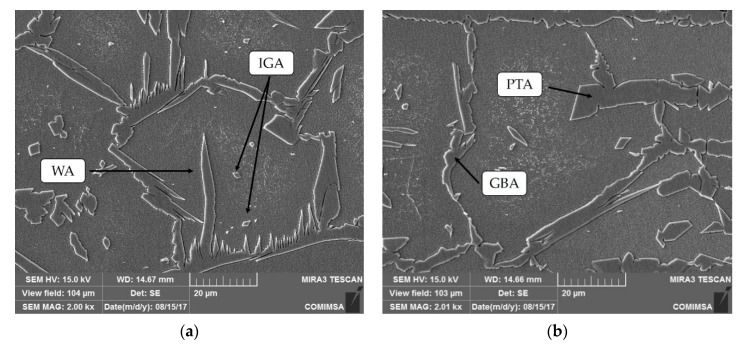
SEM micrographs of the heat-affected zone: (**a**) lean duplex stainless steel (LDSS) 2304; and (**b**) SDSS 2507. WA: Widmanstätten austenite; IGA: intragranular austenite; PTA: partially transformed austenite; GBA: grain boundary austenite.

The different types of primary austenite formed in the welded regions (Figure 9) were determined through an EDS analysis (Table 8).

**Figure 10:**
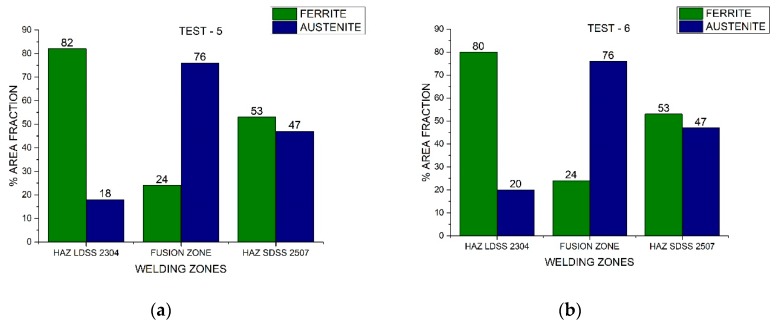
Area fraction through the distinct welded zones to 220 A: (**a**) Test #5 and (**b**) Test #6.

**Figure 11:**
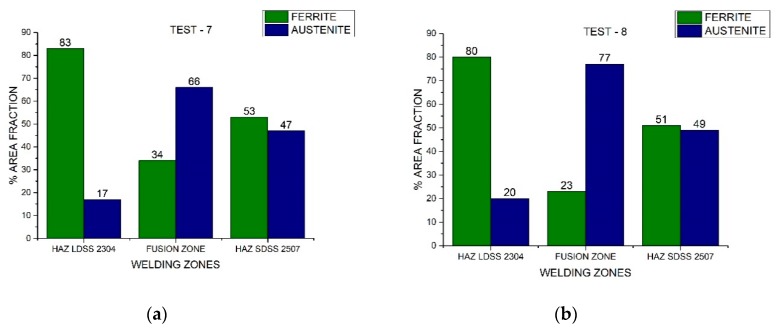
Area fraction through the distinct welded zones to 175 A: (**a**) Test #7 and (**b**) Test #8.

**Figure 12:**
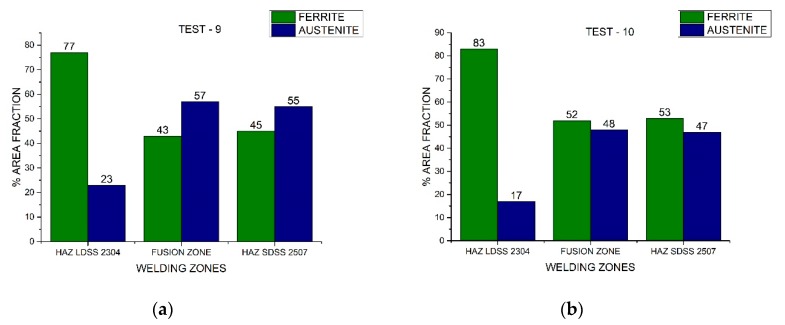
Area fraction through the distinct welded zones to 130 A: (**a**) Test #9 and (**b**) Test #10.

In Figure 10, Figure 11, and Figure 12, the area fraction of each phase in both stainless steels across the distinct welded zones was observed.

#### 3.3.2. Validation

**Figure 13:**
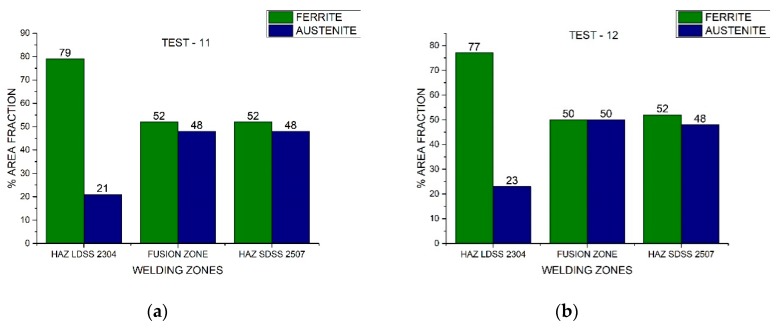
Area fraction through the distinct welded zones: (**a**) Test #11 and (**b**) Test #12.

The experimental percentage measurement of the phases in the joint matched with the information provided by the general mixed lineal model and allowed for the establishment of the process parameters, which could maintain the phase duality in the HAZ of the SDSS 2507 and the welded zone. Meanwhile, in the LDSS 2304, HAZ values near 23% of the area fraction of the austenite were shown (Figure 13).

The highest content value of austenite in the FZs and HAZs in both steels was obtained by employing a welding current of 136A and an advancing speed of 2.89 mm s^−1^.

## 4. Discussion

The initial analysis of the joint showed that the high nickel concentration in the interface of the DSS employing a welding current of 130 A was a result of the lack of heat in the mentioned zone (Figure 4), preventing the diffusion of this element through the joint. Additionally, the microstructural study revealed the formation of four different types of primary austenite in the HAZ and FZ of both steels (Figure 5 and Figure 6): grain boundary austenite (GBA), Widmanstätten austenite (WA), intragranular austenite (IGA), and partially transformed austenite (PTA) [13,24].

The effect of the filler metal was observed in Figure 5 and Figure 6, highlighting the considerable increase of the austenite as well as its coarsening in the welded zone. Furthermore, the high quantity of alloying elements that constitute the duplex stainless steel and the cooling rate allowed for the stabilization of the austenite phase during the solid-state transformation (Figure 4). The analysis through EDS (Table 8) revealed that the content of elements of the IGA was due to its precipitation in poor Cr and Mo zones and rich Ni regions, as the WA maintained minor quantities of Cr, Mo, and Ni, because its formation came later than the GBA growth (Figure 9) [13,25].

The different phase quantifications employing the process parameters generated by the design of experiments demonstrated that the microstructure depended on the thermal cycle of the welding procedure (Figure 7 and Figure 8). Different studies conducted have demonstrated that in DSS, the heat input influences the final results (which can be determined by current and speed welding), because it predicts the cooling speed value: the lower the heat input, the greater the cooling speed. The low heat input generates a high cooling speed, leading to a microstructural imbalance and promoting the excessive content of ferrite [26]. The addition of alloying elements in the shielded atmosphere or as a filler metal (i.e., nickel powder) promoted the change from ferrite to austenite at high temperatures and the later stabilization of the phase at room temperature [27].

On the other hand, the austenite ferrite dual percentage remained in the SDSS 2507 HAZ employing 220 A and 175 A in both speeds, increasing considerably the austenite quantity in the FZ (Figure 10 and Figure 11). In addition, when employing a current of 130 A, the 50/50 percentage remained in the FZ and HAZ of the SDSS 2507 (Figure 12). Nevertheless, the percentage of the austenite was reduced considerably in the HAZ of the LDSS 2304, promoting values below 25%. The optimization of the process parameters led to a welding current of 130 A and low welding speeds (Figure 13).

## 5. Conclusions

This article shows the research work carried out to analyze the influence of the GTAW process parameters on dissimilar joints of lean duplex stainless steel UNS S32404 and super duplex stainless steel UNS S32507. Based on the results provided through experimental development, the following can be concluded:

- The joints have an acceptable surface quality when using low welding current parameters (130 A) and a welding speed of 2.7 mm s^−1^.
- The autogenous GTAW process requires the amount of heat input and the cooling speed to be regulated through the welding speed and current, because a low heat input and a high cooling speed lead to an undesirable imbalance of ferrite/austenite proportion in dissimilar duplex stainless-steel joints, which affects the material properties negatively.
- The use of nickel powder as a filler metal in the GTAW process when welding duplex stainless steel favors the formation of higher content of the austenite phase in the welding zone.
- Different types of austenite (IGA, GBA, WA, and PTA) are formed in the microstructure of the joint due to the welding thermal gradients. Secondary austenite formation is not clearly identified, because the joint was not overheated because of the effect of multiple beads or subsequent heat treatments.
- These findings can be employed by welding engineers to improve the austenite/ferrite balance with nickel powder addition in the duplex stainless=steel dissimilar joints. Likely, the obtained parameters can be used as a reference.

## Figures and Tables

**Table 1:** Chemical composition of the base metals (wt %): lean duplex stainless steel (LDSS); super duplex stainless steel (SDSS).

	C	Si	Cu	Mn	Mo	Cr	Ni	S	P	N	V	W	Fe
LDSS 2304	0.02	0.48	0.29	1.37	0.36	23.5	5.02	0.014	0.031	0.10	—	—	Bal.
SDSS 2507	0.018	0.13	0.17	0.85	3.86	25.2	6.72	0.001	0.03	0.27	0.026	0.09	Bal.

**Table 2:** Chemical composition of the nickel powder (wt %).

	Ni	Cr	Cu	Si	Mg	Fe	Mn	Al	C	Ti
Nickel powder	91.73	5.28	0.005	0.56	0.002	1.04	0.008	0.036	0.22	0.015

**Table 3:** Constant parameters of the gas tungsten arc welding (GTAW) manual process.

**Tungsten Electrode**	
Classification	EWTh-2
Diameter	3.18 mm
Angle	60°
**Welding Torch**	
Angle	15° (in relation to the vertical position)
Arc length	1.58 mm
**Welding**	
Direction	Push
Position	Butt joint (plane)
Current	DCEN ^1^
Shielding gas	Ar ^2^ (15 L/min )

^1^ Direct current with a negative charged electrode; ^2^ Argon gas 5.0.

**Table 4:** Factorial design 2^k^ (preliminary test).

Test	Welding Current (A)	Nickel Powder Addition	Arc Voltage (V)
1	130	Yes	16.0
2	130	No	16.0
3	220	Yes	19.3
4	220	No	19.3

**Table 5:** Statistical analysis results. HAZ: heat-affected zone.

Zone	Parameter	P Value
HAZ 2304	Ni powder	0.961
	Welding current (A)	0.714
HAZ 2507	Ni powder	0.020
	Welding current (A)	0.029
Weld zone	Ni powder	0.022
	Welding current (A)	0.025

**Table 6:** Factorial design 2^k^ (welding parameters).

Test	Welding Current (A)	Welding Speed (mm s^−1^)	Arc Voltage (V)
5	220	3.9 (Low)	19.3
6	220	5.0 (High)	19.3
7	175	3.0 (Low))	17.2
8	175	3.4 (High)	17.2
9	130	2.8 (Low)	16.0
10	130	3.6 (High)	16.0

**Table 7:** Validation welding parameters.

Test	Welding Current (A)	Welding Speed (mm s^−1^)	Arc Voltage (V)
11	133	2.7	16.8
12	136	2.8	16.8

**Table 8:** EDS analysis (wt. %) of the phase proportions in the heat-affected zone.

Phase	LDSS 2304			SDSS 2507		
	Cr	Mo	Ni	Cr	Mo	Ni
GBA	23.18	1.26	3.80	25.09	3.09	6.55
WA	21.17	1.16	4.11	24.01	3.05	6.89
IGA	20.02	0.91	4.99	23.03	2.01	7.37
PTA	22.39	1.28	3.99	23.97	3.59	6.02
